# Variations in the application of equine prosthetic laryngoplasty: A survey of 128 equine surgeons

**DOI:** 10.1111/vsu.13913

**Published:** 2022-11-24

**Authors:** Christian A. Byrne, Joel W. Hotchkiss, Safia Z. Barakzai

**Affiliations:** ^1^ University of Glasgow Glasgow UK; ^2^ Equine Surgical Referrals Brighton UK

## Abstract

**Objective:**

To document variations in the application of equine prosthetic laryngoplasty among equine surgeons.

**Study design:**

Cross‐sectional survey.

**Sample population:**

Six hundred and seventy‐eight equine surgeons performing prosthetic laryngoplasty.

**Methods:**

An online questionnaire was sent to equine surgeons, including diplomates of the American College of Veterinary Surgeons and European College of Veterinary Surgeons. Questions focused on participant profile, surgical technique, antimicrobial therapy, and concurrent procedures. Descriptive statistical analysis was performed on the survey output.

**Results:**

Complete responses were received from 128/678 individuals, mostly from experienced surgeons. Most participants used 2 prostheses (106/128, 82.8%) and a single loop was the most common method used to anchor the prosthesis in the cricoid (95/128, 74.2%) and arytenoid (125/128, 97.7%) cartilages. Use of general anesthesia was common, although 46/128 (35.9%) participants now performed most laryngoplasty surgery with standing sedation. The material used as a prosthesis varied among surgeons, although participants typically aimed to achieve grade 2 intraoperative arytenoid abduction. Participants most commonly administered perioperative systemic antimicrobial therapy for 1‐3 days (57/128, 44.5%) and 48/128 (37.5%) used local antimicrobial therapy.

**Conclusion:**

Most surgeons performed laryngoplasty with 2 prostheses, a single loop construct at the muscular process of the arytenoid cartilage and systemic antimicrobial therapy. There was variation in the preferred method of surgical restraint, prosthesis material selection, and use of local antimicrobial therapy.

**Clinical significance:**

Long‐established techniques remain popular in clinical practice despite evidence that variations offer advantages, particularly in relation to biomechanics. Other factors are also likely to influence technique selection in a clinical context.

## 1 INTRODUCTION

Since its original description in 1970,^1^ prosthetic laryngoplasty has proved an important procedure for the management of recurrent laryngeal neuropathy.^2^ A significant body of research has investigated variations in case selection, surgical technique, and perioperative case management for prosthetic laryngoplasty. However, the popularity of these variations among equine surgeons and their use in clinical practice remains unclear. Two previous studies, performed in the early 1990s, reported the results of surveys of equine surgeons in the USA^3^ and Europe^4^ regarding the management of recurrent laryngeal neuropathy. These studies primarily focused on the diagnosis, treatment decision making, and postoperative complications associated with recurrent laryngeal neuropathy and prosthetic laryngoplasty.^3^, ^4^ It is important that even among a relatively small number of equine surgeons, some aspects of surgical technique, such as the selection of prosthesis material, varied greatly.^4^

Biomechanical attributes of the laryngoplasty construct have been a focus of recent research. Studies investigating specific components of the laryngoplasty have demonstrated benefits of variations, such as the use of a U‐shaped construct at the caudal border of the cricoid cartilage,^5^ or the use of a metallic implant in the muscular process of the arytenoid cartilage construct.^6^, ^7^, ^8^, ^9^ However, how such variations influence factors other than biomechanics, and their application in clinical practice, are poorly documented. Other anecdotal considerations, such as the use of local antimicrobial therapy in clinical cases, have not been addressed in previous literature. Gaining further understanding of potential disparities between research findings and current practice may identify potential barriers to clinical application and permit further optimization of surgical techniques.

This study aimed to survey equine surgeons on surgical technique, antimicrobial therapy, and simultaneous upper respiratory tract procedures for prosthetic laryngoplasty in a clinical context.

## 2 MATERIALS AND METHODS

### 2.1 Questionnaire

A questionnaire was prepared by the lead author using an online survey tool (Online Surveys, Jisc, Bristol, UK). The draft survey was trialed and reviewed by the coauthors, with subsequent alterations as required for the finalized version of the questionnaire, which is available in Item 1 in Appendix S1 in the supplementary material. The survey consisted of 22 questions, which included multiple choice and multiple answer (where >1 option could be selected) questions. Questions were primarily closed ended, although where appropriate, fields were included for free text comments. The survey was divided into 4 sections: participant profile, surgical technique, antimicrobial therapy, and simultaneous procedures.

The participant profile featured questions about participant speciality, the approximate number of laryngoplasty procedures performed annually, and the duration of participant experience performing laryngoplasty as the primary surgeon. The surgical technique section of the questionnaire was divided into subsections: general considerations for laryngoplasty, the cricoid, the muscular process, endoscopy, and abduction. General considerations focused on whether the surgeon primarily performed laryngoplasty with the horse under general anesthesia or standing sedation, the number of prostheses routinely placed, and the prosthesis material(s) routinely used. The cricoid section requested information regarding the degree of dissection performed at the cricoid cartilage, the technique used to anchor the prosthesis in the cricoid cartilage (including a diagram), and the needle routinely used for placement of the prosthesis in the cricoid. The section addressing the muscular process of the arytenoid documented the approach used routinely to access the muscular process, the technique used to anchor the prosthesis in the muscular process (including a diagram), and the needle routinely used for placement of the prosthesis in the muscular process. The terminology used to describe the anchoring techniques for the cricoid,^5^ and arytenoid,^6^ was based on previous literature. The endoscopy section recorded the use of intraoperative endoscopy during the placement of the prosthesis at the cricoid and when tightening the prosthesis. Where participants indicated that intraoperative endoscopy was used to assess arytenoid abduction when tightening the prosthesis, they were asked what grade of abduction they would routinely aim to achieve in a Thoroughbred racehorse and a sports horse (for example, a horse used for showjumping). A diagram was provided demonstrating the grades described by Dixon et al.,^10^ and the digital object identifier (DOI) of the reference was also supplied.

The antimicrobial therapy section of the questionnaire documented the duration of systemic antimicrobial therapy routinely used in cases undergoing laryngoplasty, the systemic antimicrobial agents routinely administered, if local antimicrobial therapy was used during laryngoplasty, and if so, how local antimicrobial therapy was administered. The section on simultaneous procedures recorded how practitioners decide which, if any other upper airway anatomical structures are operated on in combination with laryngoplasty (assuming that no previous upper respiratory tract surgery has been performed). During this section the term endoscopy was used generically, no specific reference was made to the use of resting or exercising endoscopy for case selection. If anatomical structures were routinely operated on in combination with laryngoplasty, participants were asked to select the relevant structures. Participants also reported the typical method used to perform additional procedures, for example, transendoscopically with a laser, or by laryngotomy.

### 2.2 Participant recruitment and questionnaire distribution

The online survey was distributed to participants by email. European College of Veterinary Surgeons (ECVS) specialists were contacted using email address details available online using the ECVS “Find an ECVS specialist” facility, with the large animal speciality search parameter selected (https://www.ecvs.org/animal-owners/specialist.php). American College of Veterinary Surgeon (ACVS) specialists were contacted using email address details available online on the “ACVS Diplomate Search” with the Large Animal and Equine fields included. Any individuals without an email address were not included. Any individuals present in both ECVS and ACVS databases were only contacted once. Specialists present on the ECVS or ACVS databases, without email details on the ACVS/ECVS websites, but known personally to the authors were contacted directly. Other individuals not on the ECVS or ACVS databases (including nondiplomates) but known by the authors to perform laryngoplasty surgery frequently were contacted directly. Any delivery failures following email distribution were noted. The survey was open for 3 weeks, with a reminder email sent 5 days before questionnaire closure.

### 2.3 Institutional review board ethical approval

The study was approved by the College of Medicine, Veterinary and Life Sciences Ethics Committee, University of Glasgow (project reference: 200180147). A page at the outset of the questionnaire and a participant information sheet (attached to the invitation email) outlined the purpose of the study, confidentiality considerations, and data management features. Participants were informed that participation was voluntary and that no incentive was offered for participation. The questionnaire was completed anonymously. The survey could only be completed in a single sitting; participants could not save progress and return later. Participants were informed that the questionnaire would take approximately 15 minutes to complete and that the findings of the study were intended for publication.

### 2.4 Statistical analysis

The survey output was compiled into a database for descriptive statistical analysis and graphical evaluation (Microsoft Office Excel 2016, Microsoft Corporation, Redmond, USA) (Minitab version 18.1, Minitab Ltd., Coventry, UK). Data regarding questionnaire distribution, including the number of delivery failures, were also compiled. The data were organized into frequency tables and percentages calculated as required. Graphical exploration included the formation of bar charts. For some numerical variables, analysis included calculation of the range and median.

## 3 RESULTS

### 3.1 Participants and distribution

A completed questionnaire was returned by 128 surgeons. The survey was distributed to 274 individuals from the ECVS database, 390 individuals from the ACVS database, and 14 individuals by direct contact with the authors. This resulted in an unprocessed response rate of 128/678 (18.9%). There were 27 notified email delivery failures, 10 replies from individuals outlining they were not able to complete the questionnaire (for example if they did not perform laryngoplasty surgery), and 29 out‐of‐office replies. Accounting for these distribution failures gave an adjusted response rate of 128/612 (20.9%).^11^

Surgical diplomates accounted for a large proportion of responses, with 84/128 (65.6%) participants having ACVS diplomate status and 53/128 (41.4%) having ECVS diplomate status. Postgraduate certificates (CertAVP or similar) were held by 4/128 (3.1%) participants, of which 3 participants also had ACVS/ECVS diplomate status. Other additional qualifications were reported by 6/128 (4.7%) participants. Additional qualifications included a diploma of the American College of Veterinary Sports Medicine and Rehabilitation, Royal College of Veterinary Surgeons diploma in equine soft tissue surgery, Associate Member of the European College of Veterinary Diagnostic Imaging, Fellow of the Royal College of Veterinary Surgeons, and Fellow/Member of the Australian and New Zealand College of Veterinary Scientists. A single participant (1/128, 0.8%) did not hold any additional postgraduate qualifications.

Fifty‐three participants (53/128 participants, 41.4%) reported performing approximately 1‐10 laryngoplasty procedures each year; 49/128 (38.3%) reported performing 11‐25; 15/128 (11.7%) reported performing 26‐50, and 11/128 (8.6%) reported performing >50. One participant (1/128 participants, 0.8%) reported having <1 year of experience performing laryngoplasty as the primary surgeon; 17/128 (13.3%) had 1‐5 years' experience; 29/128 (22.7%) had 6‐10 years' experience, and 81/128 (63.3%) had >10 years' experience.

### 3.2 General considerations for laryngoplasty

**FIGURE 1:**
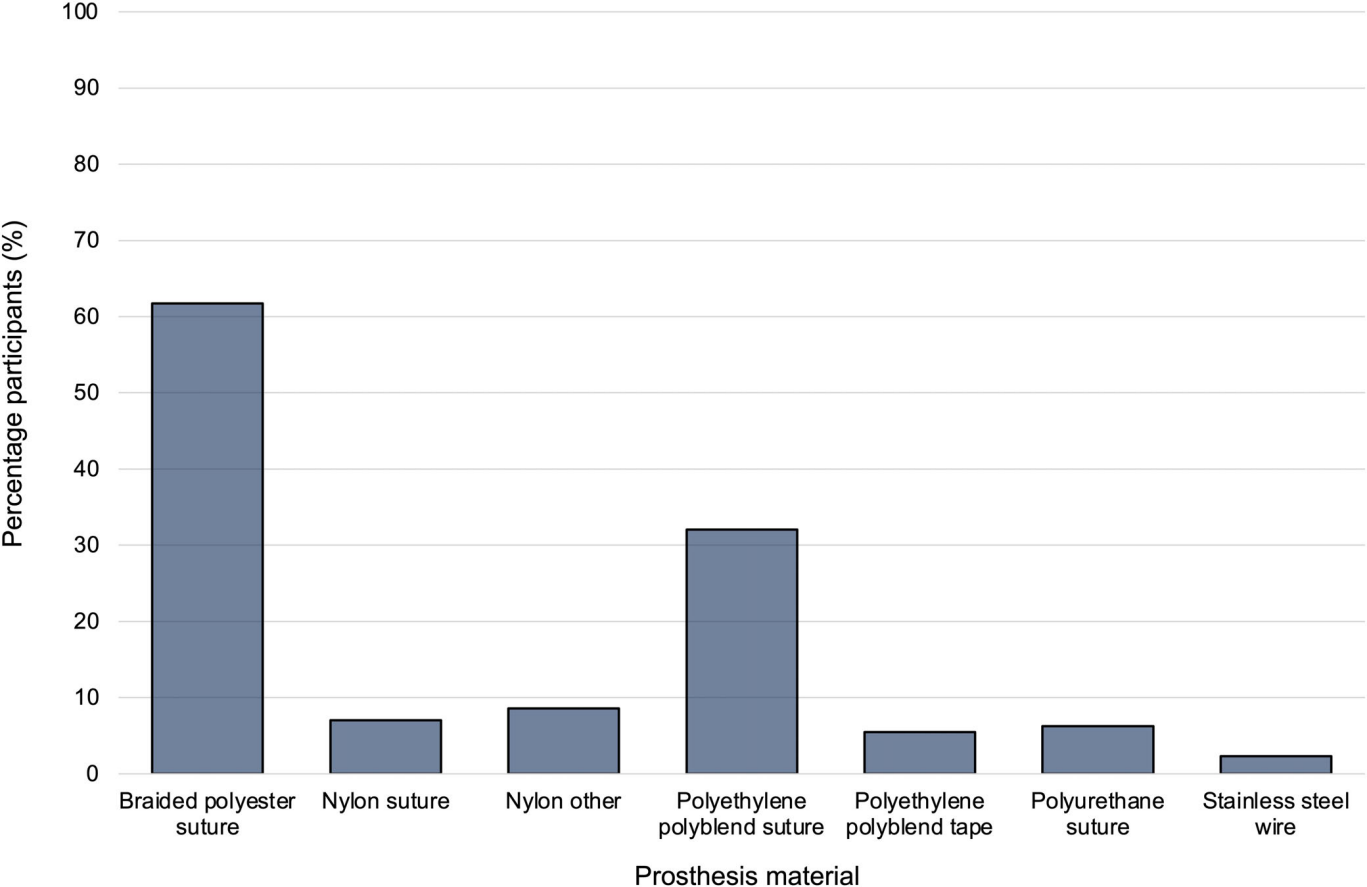
Distribution of prosthesis material routinely used for laryngoplasty by 128 equine surgeons responding to our survey

Seventy‐three participants (73/128 participants, 57.0%) routinely performed most laryngoplasty procedures under general anesthesia; 46/128 (35.9%) performed most understanding sedation, and 9/128 (7.0%) performed an equal proportion with both methods of surgical restraint. Most participants routinely placed 2 prostheses during a laryngoplasty (106/128 participants, 82.8%) and 22/128 (17.2%) placed a single prosthesis. No participants routinely placed >2 prostheses. A range of prosthesis materials were reported to be used routinely (Figure 1). A braided polyester suture was reported to be used by 79/128 (61.7%) of participants and a polyethylene polyblend suture (eg, FibreWire, Arthrex GmbH, Munich, Germany) was the second most commonly used, accounting for 41/128 (32.0%) of participants. Participants were able to select multiple materials if they used a combination, which was reported by 29/128 (22.7%) of participants.

### 3.3 Surgical technique

**FIGURE 2:**
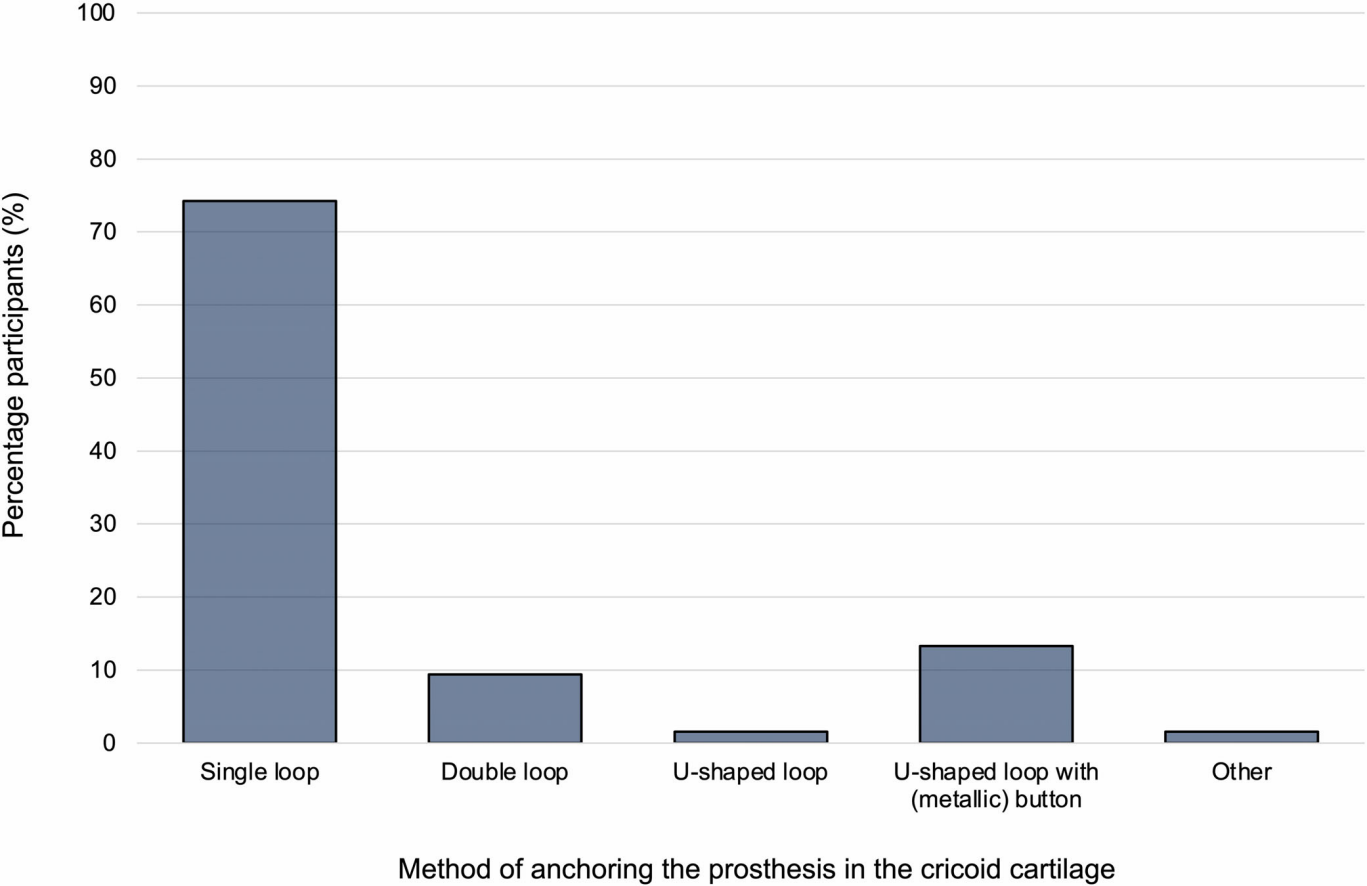
Techniques routinely used by participants to anchor the prosthesis in the cricoid cartilage during equine laryngoplasty

Seventy (70/128, 54.7%) participants dissected some tissue away from the cricoid but left a thin layer of soft tissue when the suture was placed; 43/128 (33.6%) performed minimal dissection, and 15/128 (11.7%) dissected to expose bare cartilage. Most participants (95/128 participants, 74.2%) reported placing a single loop to anchor the prosthesis in the cricoid cartilage (Figure 2). One participant reported variation in technique depending on the anatomical variation of the cricoid cartilage in individual cases. A range of needles were reported to be used for prosthesis placement in the cricoid cartilage (Table 1). A curved needle, swaged to the suture, was most common, used by 98/128 (76.6%) of participants. Participants were able to select multiple needles if they used a combination, which was reported by 9/128 (7.0%) of participants.

**TABLE 1:** Needle types reported to be routinely used to place the prosthesis in the cricoid cartilage and the muscular process of the arytenoid cartilage by 128 equine surgeons

Needle type	Count (percentage) of participants using this type of needle for prosthesis placement	
	Cricoid cartilage	Muscular process of arytenoid cartilage
Curved needle swaged to suture	98 (76.6%)	79 (61.7%)
Curved needle eyed, unswaged	29 (22.7%)	37 (28.9%)
Straight needle (eg, Jamshidi needle, hypodermic needle)	1 (0.8%)	20 (15.6%)
Deschamp's aneurysm needle	5 (3.9%)	0 (0.0%)
Passer device (eg, Scorpion)	4 (3.1%)	1 (0.8%)
Other	0 (0.0%)	1 (0.8%)

Separation of the cricopharyngeus and thyropharyngeus muscles was reported as the most common approach to the muscular process, accounting for 97/128 (75.8%) participants. Dissection caudal to the cricopharyngeus muscle was reported by 26/128 (20.3%) participants and other techniques were reported by 5/128 (3.9%). Of those answering “other,” the majority commented on using a focused dissection over the muscular process, varying technique depending on anatomical variations of the individual case, or using only limited dissection. One hundred and 25 participants (125/128 participants, 97.7%) used the single loop technique to anchor the prosthesis in the muscular process. The double loop technique, use of an implant, or another technique were reported to be used by 1 participant each. Various needles were reported to be used for prosthesis placement in the muscular process of the arytenoid cartilage (Table 1). A curved needle, swaged to the suture was reported to be the most commonly used (79/128 participants, 61.7%). Participants were able to select needles if they used a combination, which was reported by 9/128 (7.0%) of participants.

**FIGURE 3:**
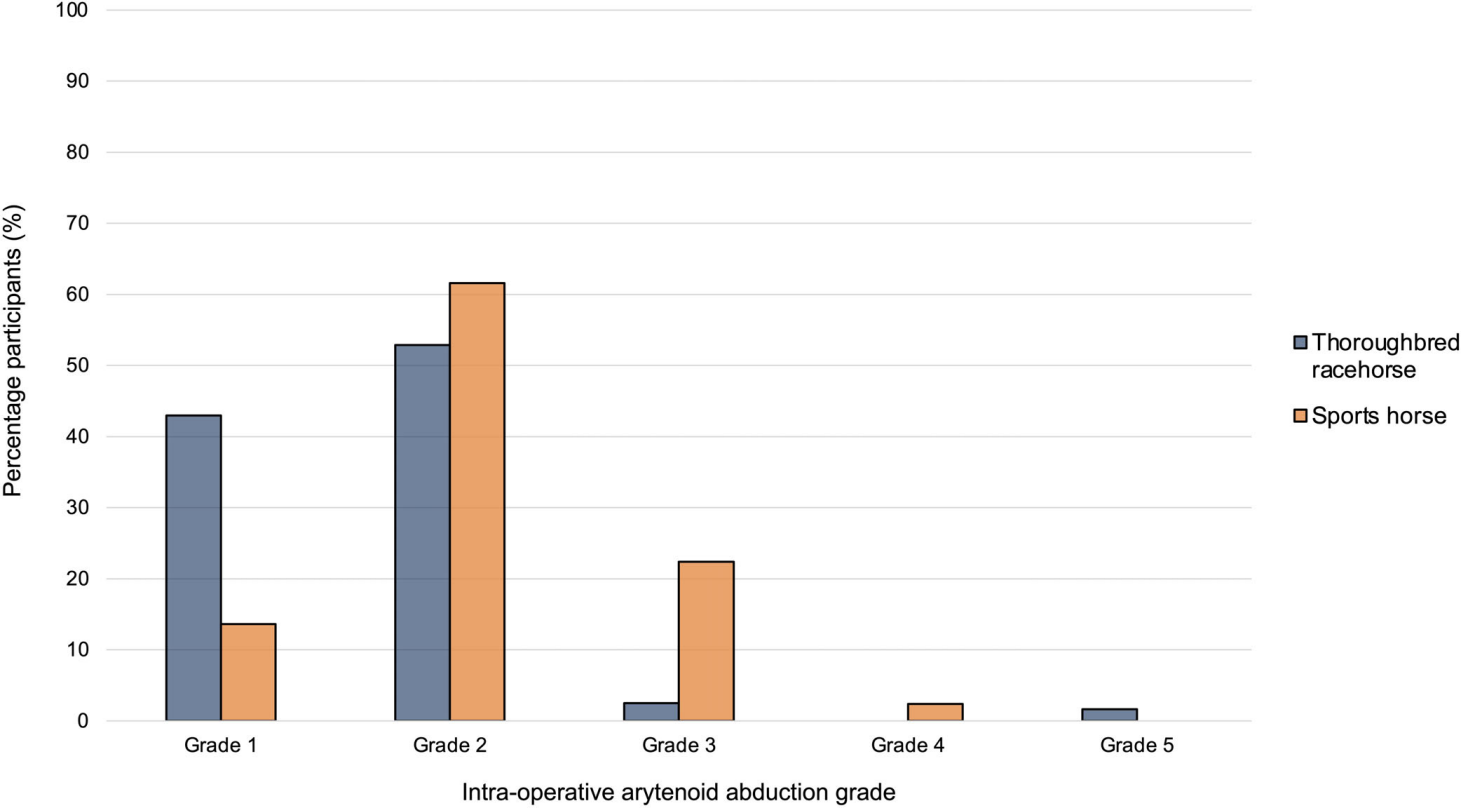
Clustered bar chart displaying the degree of intraoperative arytenoid abduction (grade) desired by participants performing laryngoplasties on Thoroughbred racehorses and sports horses

Ninety‐three participants (93/128 participants, 72.7%) routinely used intraoperative endoscopy at the time of prosthesis placement in the cricoid cartilage. One hundred and 25 participants (125/128 participants, 97.7%) used endoscopy at the time of tying or tightening the prosthesis. Some participants (3/128 participants, 2.3%) commented that assessment for luminal penetration was performed at the time of suture tying, rather than at the time of prosthesis placement. Where relevant, participants indicated the grade of intraoperative arytenoid abduction they routinely aimed to achieve (Figure 3). Grade 2 arytenoid abduction was the most common grade participants aimed to achieve for both a Thoroughbred racehorse (64/121 participants, 52.9%) and a sports horse (77/125 participants, 61.6%).

### 3.4 Antimicrobial therapy

**FIGURE 4:**
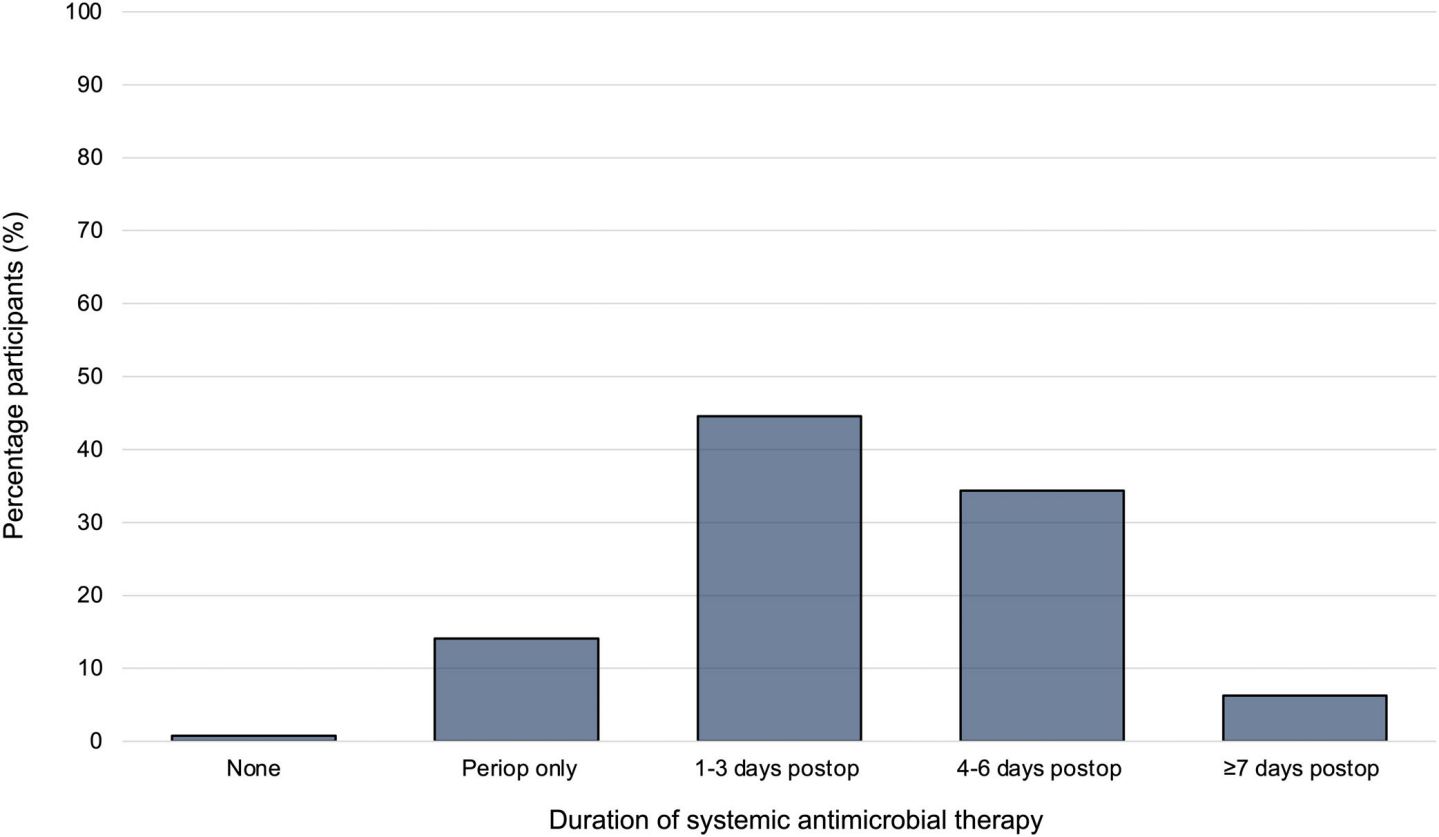
Duration of systemic antimicrobial therapy prescribed by participants performing equine laryngoplasty. Abbreviations: periop: perioperative (<24 h postoperatively), postop: postoperative

Participants most commonly administered systemic antimicrobials for 1‐3 days or 4‐6 days postoperatively (Figure 4). Of the participants that routinely administered systemic antimicrobial therapy, the most common agents were penicillin (116/127 participants, 91.3%), gentamicin (107/127 participants, 84.3%), and trimethoprim‐potentiated sulphonamides (25/127 participants, 19.7%). Other systemic agents reported to be used included oxytetracycline (2/127 participants, 1.6%) and various cephalosporin agents (3/127 participants, 2.3%). Local antimicrobial therapy was used by 48/128 (37.5%) participants. This included soaking the prosthesis in an antimicrobial agent (35/128 participants, 27.3%), lavage of the surgical site with fluid containing an antimicrobial agent (34/128 participants, 26.6%), and use of antimicrobial impregnated beads (2/128 participants, 1.6%).

### 3.5 Simultaneous procedures

**FIGURE 5:**
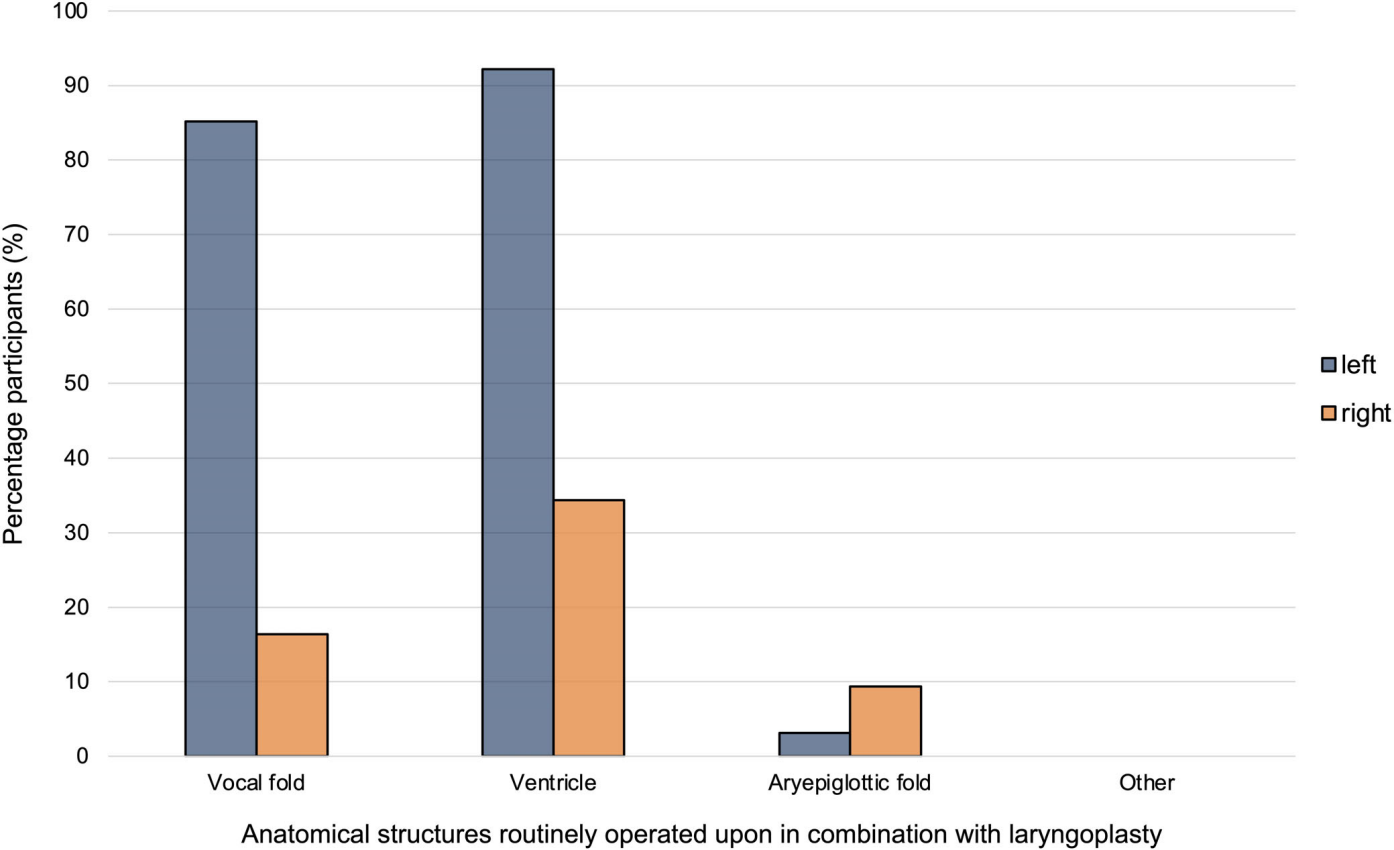
Anatomical structures routinely operated on by participants in combination with equine laryngoplasty

Forty‐eight participants (48/128 participants, 37.5%) operated on particular upper respiratory structures in combination with laryngoplasty as a routine. Sixty‐seven participants (67/128 participants, 52.3%) outlined that they routinely operated on some upper respiratory tract structures in combination with laryngoplasty but that other structures may also be operated upon based on endoscopic examination. Eleven participants (11/128 participants, 8.6%) outlined that additional procedures were only performed when there was evidence of an abnormality during endoscopy. Two participants (2/128, participants, 1.6%) commented that decision making was based on the requirement for respiratory noise reduction. The median number of structures routinely operated on in combination with a laryngoplasty was 2 (range 0‐6). The structures that participants most commonly operated on in combination with a laryngoplasty were the left ventricle (118/128 participants, 92.2%) and the left vocal fold (109/128 participants, 85.2%). Forty‐four (44/128 participants, 34.4%) routinely operated on the right ventricle (Figure 5). Transendoscopic laser surgery was the most common method for procedures to be performed in combination with laryngoplasty (89/128 participants, 69.5%), followed by laryngotomy (51/128 participants, 39.8%) and transendoscopically with electrocautery/thermocautery (4/128 participants, 3.1%). Sixteen participants (16/128 participants, 12.5%) used a combination of methods.

## 4 DISCUSSION

This study demonstrates that numerous aspects of the surgical technique proposed early in the development of laryngoplasty remain popular in clinical practice – for example, methods of anchoring the prosthesis to the cricoid and arytenoid cartilages. Many well established surgical principles, such as the use of 2 prostheses,^12^ and endoscopy at the time of prosthesis tightening, also remain commonplace. However, there is evidence of evolving case management, with a proportion of surgeons now performing the majority of laryngoplasty surgery with the horse under standing sedation rather than under general anesthesia.

The underlying principles of prosthetic laryngoplasty are clear; however, extensive literature investigating the biomechanics of the procedure introduces a more complex array of considerations for the surgeon.^13^, ^14^, ^15^ Individual variation in the morphology of the caudal border of the cricoid is well recognized^1^, ^16^ and multiple studies have investigated the interaction of the cricoid‐prosthesis construct.^5^, ^7^, ^17^ The use of a U‐shaped loop construct (particularly with the use of a ventral metallic button) is reported to have notable benefits of increased stability and avoiding the inclusion of the caudal border of the cricoid.^5^ It is therefore interesting in the present study, that most surgeons still routinely use a single loop prosthesis‐cricoid construct instead of a U‐shaped suture placement. Significant research has also evaluated the arytenoid‐prosthesis construct, including features such as loop configuration, loop orientation, needle type, and implant use.^6^, ^7^, ^17^, ^18^, ^19^ Implications of needle selection for the muscular process were evident in the present study, with a more heterogeneous distribution of selection evident for the muscular process of the arytenoid cartilage compared to that reported for the cricoid cartilage. The published literature indicates multiple potential benefits from the use of an implant (for example a metallic screw or toggle) as part of the arytenoid‐prosthesis construct;^6^, ^7^, ^8^, ^9^ however, it is evident that in the current study most surgeons still use a simple, single loop for placement of the prosthesis at the muscular process. The findings of the current study indicate that biomechanics is unlikely to be the only attribute considered by surgeons when selecting construct types in a clinical context. Other important factors may include the requirement to place 2 prostheses, prosthesis/implant availability, ease of technique, differences in personnel or equipment requirements, cost, and risks of biomechanical or nonbiomechanical complications (for example, differences in risk of implant infection between constructs).

A suitable surgical approach and level of dissection are important to permit satisfactory placement of the prosthesis. In the current study, a range of dissection techniques to expose the caudal border of the cricoid were reported by surgeons, which may be a manifestation of surgeon preference; increased dissection close to the cartilage at this site reveals large vessels such as the cranial thyroid artery, caudal laryngeal artery and associated veins, which if damaged can cause intraoperative bleeding that can significantly impact visualization.^20^, ^21^, ^22^ Different dissection preferences may also reflect variation between case populations and disparities in dissection requirements between prosthesis constructs. It is now well recognized that the esophageal diverticulum is an important structure to avoid during prosthesis placement in the muscular process of the arytenoid cartilage.^23^, ^24^ Dissection between the cricopharyngeus and thyropharyngeus muscles allows clear identification and retraction of the esophageal diverticulum^24^ and this was the preferred method to expose the muscular process in the current survey.

Biomechanical research has also been important in relation to the selection of appropriate prosthesis materials.^9^, ^13^, ^14^, ^15^ The original description of the prosthetic laryngoplasty in 1970 utilized a polyurethane suture,^1^ although a later survey in 1993 indicated that surgeons at that time elected to use a variety of other prosthesis materials.^4^ Subsequent literature has continued to demonstrate the use of a large variety of different prosthesis materials in clinical practice.^1^, ^10^, ^25^, ^26^, ^27^, ^28^, ^29^, ^30^, ^31^, ^32^, ^33^ This was mirrored in the findings of the current study, with a multitude of materials reported to be used. However, braided polyester and polyethylene polyblend sutures accounted for the vast majority of participants. This diversity reflects the multifactorial decision making in the selection of prosthesis material, which likely encompasses biomechanics (including elasticity), handling properties, cost, availability, compatibility with anchoring methods, tissue reaction, and perceived risk of infection.

Tying the prosthesis (or otherwise securing the prosthesis by another method) is an important step in the laryngoplasty. Endoscopy is recommended for intraoperative assessment of abduction^15^ and was reported to be used by almost all participants in the current study. Previous literature has documented that there is typically some loss of abduction postoperatively following laryngoplasty.^3^, ^10^, ^21^, ^34^ Conversely, complications can also be associated with excessive abduction.^10^, ^15^, ^21^ Surgeons must therefore decide and execute the desired degree of arytenoid abduction for the individual case during surgery. Dixon grade 2 intraoperative abduction is typically deemed to be an appropriate intraoperative aim;^10^, ^15^, ^35^ however, there is limited evidence to document the clinical application of this by equine surgeons. In the present study, Dixon grade 2 was indeed the most commonly selected intraoperative abduction that surgeons aimed to achieve, although there was a trend towards greater abduction if the case was a Thoroughbred racehorse and less abduction for a sports horse (eg, a horse used for showjumping). Simultaneous surgical procedures performed on other structures of the upper respiratory tract were also commonly reported in the current survey. Though preoperative diagnostic findings may influence the additional upper respiratory tract structures operated on in an individual case, the current study indicates that many surgeons operate on some structures as a routine. Previous evidence indicates that left ventriculocordectomy before laryngoplasty is advantageous^36^ and, in the current study, most surgeons reported routine removal of the left ventricle and left vocal fold. Fewer surgeons routinely operated on right‐sided structures (compared to those of the left), except for the right aryepiglottic fold.^31^, ^37^ This is likely a manifestation of previous publications, which found that right‐sided medial deviation of the aryepiglottic fold is commonly associated with grade C/D laryngeal abduction at exercise and also after left‐sided prosthetic laryngoplasty.^31^, ^34^, ^35^ Many surgeons reported that they performed simultaneous upper respiratory tract procedures transendoscopically using a laser, although sharp dissection through a laryngotomy also remains a popular approach. Both methods have potential advantages and disadvantages,^21^, ^38^, ^39^ and the differences in chosen approach likely reflect surgeon preference and availability of transendoscopic laser facilities.

Prosthetic laryngoplasty is classified as a clean surgical wound, so surgical site infection is expected to be uncommon, with rates of 0%‐4% reported.^40^ However, when they occur, surgical site and/or implant infection are serious complications.^15^, ^21^, ^41^ It is typical for perioperative antimicrobial therapy to be used in cases undergoing prosthetic laryngoplasty.^15^ Previous reports demonstrate notable variation in the antimicrobial agents utilized and the typical duration of administration,^1^, ^25^, ^26^, ^27^, ^29^, ^31^, ^33^, ^42^ but there is a lack of literature evaluating this specifically. The current study indicates that penicillin and gentamicin were the most utilized agents, with surgeons typically administering systemic antimicrobials for 1‐3 days postoperatively. Local antimicrobial therapy has not been evaluated in previous laryngoplasty surveys,^3^, ^4^ but the current study reveals that this was employed by 48/128 (37.5%) participants, with roughly equal proportions soaking the prosthesis or lavaging the surgical site with fluid that contained an antimicrobial agent. A small number of participants also reported the use of absorbable antimicrobial‐impregnated beads. Effective local antimicrobial therapy may reduce the use of systemic antimicrobial therapy.^40^ However, care must be taken to ensure that administration is appropriate and does not promote the development of antimicrobial resistance (such as exposure of pathogens to subtherapeutic levels of an agent in lavage fluids).^43^ Further investigation of methods of local antimicrobial therapy and the influence of other factors (such as prosthesis material) on surgical site infection in equine laryngoplasty is warranted. This research may be translated to alternative procedures also used for the management of recurrent laryngeal neuropathy such as dynamic neuroprosthesis^44^ and nerve transplantation,^15^, ^45^, ^46^ which have some similar considerations.

The standing laryngoplasty procedure was initially developed to avoid the requirement of general anesthesia in large draft horses that may be at greater risk of anesthesia‐related complications.^33^ However, the avoidance of anesthesia and reported improvement in the intraoperative assessment of arytenoid abduction^15^ had perceived benefits to other breeds, resulting in application to a greater population of horses.^33^ Given the relatively recent description of this technique, it is interesting that 46/128 (35.9%) participants now performed most laryngoplasty understanding sedation in the current study. The proportion of laryngoplasty procedures performed under standing sedation is likely to increase in the future due to the adoption of the standing procedure by more surgeons and further owner awareness that standing surgery is often an appropriate option.

Variation in surgeon preference for certain aspects of laryngoplasty in the present study provides valuable opportunities to optimize surgical technique. The survey findings indicate some disparity in the adoption of apparently beneficial variations by surgeons in clinical practice. For example, there has been a relatively prompt transition to the use of laryngoplasty in standing horses, but other variations, including some of those demonstrated to provide a biomechanical advantage, appear to have lower adoption. This discrepancy may reflect the ease with which a variation in technique may be adopted, intrinsic limitations of studies focusing on specific aspects of the procedure, and the influence of other factors in clinical practice, such as consumable availability and economics. Where uptake of a potentially advantageous variation has been limited, additional evidence of clinical application and outcomes may be valuable. Furthermore, the current study indicates that surgical site and/or prosthesis infection remain important considerations, but evidence to guide therapy selection is limited. Further investigation to characterize typical bacterial isolates (from surgical site and implant infections) and evaluate the potential efficacy of local antimicrobial therapy would therefore be valuable.

The current study had limitations, some of which were a result of the survey‐based method. Our adjusted response rate of 20.9% is consistent with that reported in other recent surveys of equine veterinary specialists.^47^, ^48^, ^49^, ^50^ Nonetheless, the survey response rates are likely to represent an underestimate of the true response rate for eligible participants (true response rate = number of responses/contacted individuals who perform laryngoplasty surgery). Those receiving the invitation email were asked to self‐select as practitioners performing laryngoplasty surgery. An unknown proportion of email recipients (who do not perform laryngoplasty surgery) therefore contributed to the response rate denominators reported above but would not have been eligible to participate. There is also likely to be some bias in the participant population, relative to the true population of practitioners performing laryngoplasty in clinical practice. The distribution methods may introduce a bias for participants to be diploma holders and the nature of the survey may stimulate a bias for those with a specific interest in upper respiratory tract surgery to be more likely to respond. Combining the relatively low response rate with the potential overrepresentation of diploma holders, experienced surgeons, or those with a specific interest in respiratory surgery is likely to introduce some bias relative to the population of surgeons performing laryngoplasty in clinical practice. For example, those with a specific interest in respiratory surgery may have a bias towards adopting reported variations in laryngoplasty technique compared to those only performing the procedure infrequently. The survey focused on certain key aspects of laryngoplasty, to ensure that it did not become prohibitively long. As a result, some relevant topics were not covered, such as case selection decision making, surgical interventions related to the crico‐arytenoid articulation, and management of specific complications. Optional free text response fields were included but a survey of this nature provides limited information about the reasoning behind participant decision making. Complementary study methods such as focus group discussions could provide further clarification on the underlying reasons for variation in surgical technique.

In conclusion, conventional techniques such as the use of 2 prostheses, with a single loop construct at the cricoid and arytenoid remain popular with many surgeons. There is evidence of active development of the procedure, exemplified by the trend towards standing laryngoplasty. Previous literature has demonstrated potential benefits of variations concerning particular attributes or constructs (for example a biomechanical advantage) but the persistence of some long‐established techniques indicates that other factors are likely to influence surgeon decision making and technique selection.

## CONFLICT OF INTEREST

The authors declare no conflicts of interest related to this report.

## Supporting information


**Appendix S1:** Survey questions forming the questionnaire

## References

[1] Marks D , Mackay‐Smith MP , Cushing LS , Leslie JA . Use of a prosthetic device for surgical correction of laryngeal hemiplegia in horses. J Am Vet Med Assoc. 1970;157(2):157‐163. 5463883

[2] Parente EJ . Fifty years of recurring struggles with recurrent laryngeal neuropathy. Equine Vet J. 2018;50(2):155‐158. 28976020 10.1111/evj.12763

[3] Sweeney CR . Left laryngeal hemiplegia in the horse: a survey of diagnostic criteria and management practices employed by 25 veterinarians in the United States. Equine Vet Educ. 1992;4(2):93‐95.

[4] Bathe AP . Left laryngeal hemiplegia in the horse: a survey of diagnostic criteria and management practices employed by 20 veterinary surgeons in Europe. Equine Vet Educ. 1993;5(2):84‐85.

[5] Brandenberger O , Rossignol F , Perkins JD , et al. Ex vivo biomechanical stability of 5 cricoid‐suture constructs for equine laryngoplasty. Vet Surg. 2017;46(5):705‐713. 28498552 10.1111/vsu.12671

[6] Lechartier A , Rossignol F , Brandenberger O , et al. Mechanical comparison of 3 anchoring techniques in the muscular process for Laryngoplasty in the equine larynx. Vet Surg. 2015;44(3):333‐340. 25069790 10.1111/j.1532-950X.2014.12248.x

[7] Ahern BJ , Boston RC , Parente EJ . In vitro mechanical testing of an alternate Laryngoplasty system(ALPS) for horses. Vet Surg. 2012;41(8):918‐923. 23198920 10.1111/j.1532-950X.2012.01061.x

[8] Ahern BJ , van Eps AW , Boston RC , Franklin SH . In vitro comparison of 3 techniques of prosthesis attachment to the muscular process of the equine arytenoid cartilage. Vet Surg. 2017;46(5):700‐704. 28462516 10.1111/vsu.12659

[9] Pressanto MC , Pascoletti G , Perkins JD , et al. Ex vivo biomechanical evaluation of polyester and polyblend suture techniques to perform equine laryngoplasty. Vet Surg. 2022;51(3):497‐508. 35129218 10.1111/vsu.13777

[10] Dixon PM , McGorum BC , Railton DI , et al. Long‐term survey of laryngoplasty and ventriculocordectomy in an older, mixed‐breed population of 200 horses. Part 1: maintenance of surgical arytenoid abduction and complications of surgery. Equine Vet J. 2010;35(4):389‐396. 10.2746/042516403776014172 12880007

[11] Christley RM . Questionnaire survey response rates in equine research. Equine Vet J. 2016;48(2):138‐139. 26820584 10.1111/evj.12552

[12] Kelly JR , Carmalt J , Hendrick S , Wilson DG , Shoemaker R . Biomechanical comparison of six suture configurations using a large diameter polyester prosthesis in the muscular process of the equine arytenoid cartilage. Vet Surg. 2008;37(6):580‐587. 19134109 10.1111/j.1532-950X.2008.00423.x

[13] Ahern BJ , Parente EJ . Mechanical evaluation of the equine laryngoplasty. Vet Surg. 2010;39(6):661‐666. 20459484 10.1111/j.1532-950X.2010.00701.x

[14] Ahern BJ . Biomechanics of prosthetic Laryngoplasty. In: Hawkins J , ed. Advances in Equine Upper Respiratory Surgery. Vol 24. John Wiley & Sons, Inc.; 2015:35‐41.

[15] Ducharme NG , Rossignol F . Chapter 46 ‐ larynx. In: Auer JA , Stick JA , Kümmerle JM , Prange T , eds. Equine Surgery. 5th ed. W.B. Saunders; 2019:734‐769.

[16] Dahlberg JA , Valdes‐Martinez A , Boston RC , Parente EJ . Analysis of conformational variations of the cricoid cartilages in thoroughbred horses using computed tomography. Equine Vet J. 2011;43(2):229‐234. 21592220 10.1111/j.2042-3306.2010.00070.x

[17] Schumacher J , Wilson AM , Pardoe C , Easter JL . In vitro evaluation of a novel prosthesis for laryngoplasty of horses with recurrent laryngeal neuropathy. Equine Vet J. 2000;32(1):43‐46. 10661384 10.2746/042516400777611991

[18] Rossignol F , Perrin R , Desbrosse F , Elie C . In vitro comparison of two techniques for suture prosthesis placement in the muscular process of the equine arytenoid cartilage. Vet Surg. 2006;35(1):49‐54. 16409409 10.1111/j.1532-950X.2005.00111.x

[19] Secor EJ , Gutierrez‐Nibeyro SD , Horn GP . Biomechanical evaluation of modified laryngoplasty by use of a toggle technique for stabilization of arytenoid cartilage in specimens obtained from equine cadavers. Am J Vet Res. 2018;79(2):226‐232. 29359970 10.2460/ajvr.79.2.226

[20] Ahern BJ , Parente EJ . Surgical complications of the equine upper respiratory tract. Vet Clin North Am Equine Pract. 2008;24(3):465‐484. 19203696 10.1016/j.cveq.2008.10.004

[21] Froydenlund TJ , Dixon PM . A review of equine laryngoplasty complications. Equine Vet Educ. 2014;26(2):98‐106.

[22] Rossignol F , Ducharme NG . Complications in larynx surgery. In: Rubio‐Martinez LM , Hendrickson DA , eds. Complications in Equine Surgery. Vol. 2021. John Wiley & Sons, Inc.; 438‐467.

[23] Barakzai SZ , Dixon PM , Hawkes CS , Cox A , Barnett TP . Upper esophageal incompetence in five horses after prosthetic Laryngoplasty. Vet Surg. 2015;44(2):150‐155. 24484183 10.1111/j.1532-950X.2014.12101.x

[24] Brandenberger O , Martens A , Robert C , et al. Anatomy of the vestibulum esophagi and surgical implications during prosthetic laryngoplasty in horses. Vet Surg. 2018;47(7):942‐950. 30230559 10.1111/vsu.12944

[25] Hawkins JF , Tulleners EP , Ross MW , Evans LH , Raker CW . Laryngoplasty with or without ventriculectomy for treatment of left laryngeal hemiplegia in 230 racehorses. Vet Surg. 1997;26(6):484‐491. 9387213 10.1111/j.1532-950x.1997.tb00521.x

[26] Kidd JA , Slone DE . Treatment of laryngeal hemiplegia in horses by prosthetic laryngoplasty, ventriculectomy and vocal cordectomy. Vet Rec. 2002;150(15):481‐484. 11995680 10.1136/vr.150.15.481

[27] Kraus BM , Parente EJ , Tulleners EP . Laryngoplasty with ventriculectomy or ventriculocordectomy in 104 draft horses (1992‐2000). Vet Surg. 2003;32(6):530‐538. 14648531 10.1111/j.1532-950x.2003.00530.x

[28] Scherzer S , Hainisch EK . Evaluation of a canine cranial cruciate ligament repair system for use in equine laryngoplasty. Vet Surg. 2005;34(6):548‐553. 16343140 10.1111/j.1532-950X.2005.00086.x

[29] Barakzai SZ , Boden LA , Dixon PM . Race performance after laryngoplasty and ventriculocordectomy in National Hunt racehorses. Vet Surg. 2009;38(8):941‐945. 20017851 10.1111/j.1532-950X.2009.00600.x

[30] Witte TH , Mohammed HO , Radcliffe CH , Hackett RP , Ducharme NG . Racing performance after combined prosthetic laryngoplasty and ipsilateral ventriculocordectomy or partial arytenoidectomy: 135 thoroughbred racehorses competing at less than 2400 m (1997‐2007). Equine Vet J. 2009;41(1):70‐75. 19301585 10.2746/042516408x343163

[31] Leutton JL , Lumsden JM . Dynamic respiratory endoscopic findings pre‐ and post laryngoplasty in thoroughbred racehorses. Equine Vet J. 2015;47(5):531‐536. 25124793 10.1111/evj.12331

[32] Raffetto JA , Wearn JG , Fischer AT Jr . Racing performance following prosthetic laryngoplasty using a polyurethane prosthesis combined with a laser‐assisted ventriculocordectomy for treatment of recurrent laryngeal neuropathy in 78 thoroughbred racehorses. Equine Vet J. 2015;47(1):60‐64. 25679021 10.1111/evj.12237

[33] Rossignol F , Vitte A , Boening J , et al. Laryngoplasty in standing horses. Vet Surg. 2015;44(3):341‐347. 25864499 10.1111/vsu.12307

[34] Barnett TP , O'Leary JM , Parkin TDH , Dixon PM , Barakzai SZ . Long‐term maintenance of arytenoid cartilage abduction and stability during exercise after Laryngoplasty in 33 horses. Vet Surg. 2013;42(3):291‐295. 23452305 10.1111/j.1532-950X.2013.01109.x

[35] Rakesh V , Ducharme NG , Cheetham J , Datta AK , Pease AP . Implications of different degrees of arytenoid cartilage abduction on equine upper airway characteristics. Equine Vet J. 2008;40(7):629‐635. 19165931 10.2746/042516408x330329

[36] Perkins JD , Meighan H , Windley Z , Troester S , Piercy R , Schumacher J . In vitro effect of Ventriculocordectomy before Laryngoplasty on abduction of the equine arytenoid cartilage. Vet Surg. 2011;40:305‐310. 21314703 10.1111/j.1532-950X.2011.00796.x

[37] King DS , Tulleners E , Martin BB Jr , Parente EJ , Boston R . Clinical experiences with axial deviation of the aryepiglottic folds in 52 racehorses. Vet Surg. 2001;30(2):151‐160. 11230769 10.1053/jvet.2001.21389

[38] Henderson CE , Sullins KE , Brown JA . Transendoscopic, laser‐assisted ventriculocordectomy for treatment of left laryngeal hemiplegia in horses: 22 cases (1999‐2005). J Am Vet Med Assoc. 2007;231(12):1868‐1872. 18081528 10.2460/javma.231.12.1868

[39] Cramp P , Barakzai SZ . Surgical management of recurrent laryngeal neuropathy. Equine Vet Educ. 2012;24(6):307‐321.

[40] Stewart S , Richardson DW . Chapter 7 ‐ surgical site infection and the use of antimicrobials. In: Auer JA , Stick JA , Kümmerle JM , Prange T , eds. Equine Surgery. Fifth ed. W.B. Saunders; 2019:77‐103.

[41] Fitzharris LE , Lane JG , Allen KJ . Outcomes of horses treated with removal of a laryngoplasty prosthesis. Vet Surg. 2019;48(4):465‐472. 30609094 10.1111/vsu.13150

[42] Mason BJ , Riggs CM , Cogger N . Cohort study examining long‐term respiratory health, career duration and racing performance in racehorses that undergo left‐sided prosthetic laryngoplasty and ventriculocordectomy surgery for treatment of left‐sided laryngeal hemiplegia. Equine Vet J. 2013;45(2):229‐234. 22812572 10.1111/j.2042-3306.2012.00601.x

[43] Isgren CM . Improving clinical outcomes via responsible antimicrobial use in horses. Equine Vet Educ. 2022;34:482‐492. doi:10.1111/eve.13502

[44] Rossignol F , Ducharme N . Reinnervation of the larynx: where are we in 2021? 30th Annual Scientific Meeting of the European College of Veterinary Surgeons; European College of Veterinary Surgeons, 2021.

[45] Fulton IC , Derksen FJ , Stick JA , Robinson NE , Duncan ID . Histologic evaluation of nerve muscle pedicle graft used as a treatment for left laryngeal hemiplegia in standardbreds. Am J Vet Res. 1992;53(4):592‐596. 1586034

[46] Rossignol F , Brandenberger O , Perkins JD , Marie JP , Mespoulhès‐Rivière C , Ducharme NG . Modified first or second cervical nerve transplantation technique for the treatment of recurrent laryngeal neuropathy in horses. Equine Vet J. 2018;50(4):457‐464. 29193393 10.1111/evj.12788

[47] Lefebvre D , Pirie RS , Handel IG , Tremaine WH , Hudson NPH . Clinical features and management of equine post operative ileus: survey of diplomates of the European colleges of equine internal medicine (ECEIM) and veterinary surgeons (ECVS). Equine Vet J. 2016;48(2):182‐187. 25256601 10.1111/evj.12355

[48] Lefebvre D , Hudson NPH , Elce YA , et al. Clinical features and management of equine post operative ileus (POI): survey of diplomates of the American colleges of veterinary internal medicine (ACVIM), veterinary surgeons (ACVS) and veterinary emergency and critical care (ACVECC). Equine Vet J. 2016;48(6):714‐719. 26502215 10.1111/evj.12520

[49] Lawson AL , Sherlock CE , Ireland JL , Mair TS . Equine nutrition in the post‐operative colic: survey of diplomates of the American colleges of veterinary internal medicine and veterinary surgeons, and European colleges of equine internal medicine and veterinary surgeons. Equine Vet J. 2021;53(5):1015‐1024. 33174212 10.1111/evj.13381 PMC8451781

[50] Knott LE , Fonseca‐Martinez BA , O'Connor AM , Goodrich LR , McIlwraith CW , Colbath AC . Current use of biologic therapies for musculoskeletal disease: a survey of board‐certified equine specialists. Vet Surg. 2022;51:557‐567. doi:10.1111/vsu.138051 35383972 PMC9322007

