# Supplementary material for: Variations in the application of equine prosthetic laryngoplasty: A survey of 128 equine surgeons
Source: Vet Surg. 2022 Nov 24;52(2):209–20. doi: 10.1111/vsu.13913 (PMC10100511; doi:10.1111/vsu.13913)
Supplement: Supplementary file 1 — Appendix S1: Survey questions forming the questionnaire [file VSU-52-209-s001.docx]

**Supplementary Item 1.** Survey questions forming the questionnaire

**Participant profile**

1. What is your current speciality status? (select all that apply)
   1. No specialist qualifications
   2. Certificate holder (CertAVP or similar)
   3. Diplomate- ECVS
   4. Diplomate- ACVS
   5. MANZCVS or FANZCVS
   6. Other
2. Approximately how many prosthetic laryngoplasty procedures do you perform each year?
   1. 1-10
   2. 11-25
   3. 26-50
   4. >50
3. Please select the duration of your experience performing laryngoplasty procedures as the primary surgeon?
   1. <1 year
   2. 1-5 years
   3. 6-10 years
   4. >10 years
4. Any additional comments

**General considerations for laryngoplasty**

1. Please indicate the proportion of laryngoplasty procedures you currently perform under general anaesthesia (GA) or standing sedation
   1. Majority under GA
   2. An equal proportion under GA and standing sedation
   3. Majority under standing sedation
2. How many prostheses do you routinely place during a laryngoplasty?
   1. One
   2. Two
   3. Other
3. What prosthesis material do you routinely use? (select all that apply for combinations)
   1. Braided polyester suture (e.g. Ethibond, Ti-Cron)
   2. Nylon suture (e.g. Ethilon)
   3. Nylon other (e.g. Securos)
   4. Polyethylene polyblend suture (e.g. FiberWire)
   5. Polyethylene polyblend tape (e.g. FiberTape)
   6. Polyurethane suture (e.g. Lycra)
   7. Stainless steel wire
   8. Other
4. Any additional comments

**The cricoid**

1. How much dissection do you routinely perform at the cricoid cartilage?
   1. I dissect soft tissue until I can visualise the cartilage and suture is placed onto the bare cartilage
   2. I dissect some tissue away from the cricoid but there is still a thin layer of soft tissue when I place the suture
   3. I perform minimal dissection at the cricoid cartilage
2. What technique do you routinely use to anchor the prosthesis to the cricoid cartilage? See below for diagramtic representations of constructs (dorsal view)
   1. Single loop
   2. Double loop
   3. U-shaped loop
   4. U-shaped loop with (metallic) button
   5. Other
3. What variety of needle do you routinely use to place the prosthesis in the cricoid? (select all that apply).
   1. Curved needle- swaged to suture
   2. Curved needle- eyed, unswaged
   3. Straight needle (e.g. Jamshidi needle, hypodermic needle)
   4. Deschamp’s aneurysm needle
   5. Other suture passer (e.g. Scorpion)
   6. Other
4. Any additional comments

**The muscular process**

1. Which approach do you routinely use to access the muscular process?
   1. Separate between the cricopharyngeus and thyropharyngeus muscles
   2. Dissect cadual to the cricopharyngeus (retract the cricopharyngeus rostrally)
   3. Other
2. Which technique do you routinely use to anchor the prosthesis to the muscular process of the arytenoid (select all that apply if you use multiple prostheses)? See below for diagramatic representations of constructs (dorsal view).
   1. Single loop
   2. Double loop
   3. Implant (e.g. toggle, screw or button)
   4. Other
3. What variety of needle do you routinely use to place the prosthesis in the muscular process of the arytenoid? (select all that apply).
   1. Curved needle- swaged to suture
   2. Curved needle- eyed, unswaged
   3. Straight needle (e.g. Jamshidi needle, hypodermic needle)
   4. Deschamp’s aneurysm needle
   5. Passer device (e.g. Scorpion)
   6. Other
4. Any additional comments

**Endoscopy and abduction**

1. Do you routinely use intra-operative endoscopic guidance when placing suture at cricoid?
   1. Yes
   2. No
2. Do you routinely use intra-operative endoscopic guidance when tightening and tying the prosthesis?
   1. Yes
   2. No
3. Any additional comments
4. What grade of arytenoid abduction would you routinely aim to achieve intra-operatively for a Thoroughbred racehorse? (see below for Dixon et al. (2003) grading system)
   1. Grade 1
   2. Grade 2
   3. Grade 3
   4. Grade 4
   5. Grade 5
5. What grade of arytenoid abduction would you routinely aim to achieve intra-operatively for a sports horse (e.g. used for jumping)? (see below for Dixon et al. (2003) grading system)
   1. Grade 1
   2. Grade 2
   3. Grade 3
   4. Grade 4
   5. Grade 5
6. Any additional comments

**Antimicrobial therapy**

1. What duration of systemic antimicrobial therapy do you routinely prescribe for a laryngoplasty?
   1. None
   2. Peri-operative only
   3. 1-3 days postoperatively
   4. 4-6 days postoperatively
   5. ≥7 days postoperatively
2. Which systemic antimicrobial agents do you routinely use in the perioperative period for a laryngoplasty procedure? (select all that apply)
   1. Penicillin
   2. Gentamicin
   3. Oxytetracycline
   4. Trimethoprim-potentiated sulphonamides
   5. Other
3. Do you routinely use local antimicrobial therapy during the laryngoplasty procedure? (select all that apply)
   1. Yes- prosthesis material soaked in antimicrobial medication
   2. Yes- surgical site lavaged with antimicrobial (including if diluted in lavage fluids)
   3. No
   4. Other
4. Any additional comments

**Simultaneous procedures**

1. If you perform any additional upper airway procedures in combination with laryngoplasty (e.g. ventriculectomy, vocal cordectomy), how do you decide which structures are operated? Assume that no previous laryngeal surgery has been performed.
   1. Particular structures are operated as a routine
   2. Particular structures are routinely operated, but additional procedures may be performed based on endoscopic (resting or dynamic) examination
   3. Structures are always selected based on evidence of abnormality during endoscopic (resting or dynamic) examination
   4. Other
2. If relevant, please indicate the structures you routinely operate on in combination with a laryngoplasty, assuming that none of these structures have previously been operated upon. (select all that apply)
   1. Left vocal fold
   2. Left ventricle
   3. Left aryepiglottic fold
   4. Right vocal fold
   5. Right ventricle
   6. Right aryepiglottic fold
   7. Soft palate
   8. Other
3. If you perform additional laryngeal procedures in combination with the laryngoplasty, how is this typically performed? (select all that apply)
   1. By laryngotomy
   2. Transendoscopically with a laser.
   3. Other
4. Any additional comments
